# Carbonic Anhydrase I as a New Plasma Biomarker for Prostate Cancer

**DOI:** 10.5402/2012/768190

**Published:** 2012-11-19

**Authors:** Michiko Takakura, Akira Yokomizo, Yoshinori Tanaka, Michimoto Kobayashi, Giman Jung, Miho Banno, Tomohiro Sakuma, Kenjiro Imada, Yoshinao Oda, Masahiro Kamita, Kazufumi Honda, Tesshi Yamada, Seiji Naito, Masaya Ono

**Affiliations:** ^1^Division of Chemotherapy and Clinical Research, National Cancer Center Research Institute, 5-1-1 Tsukiji, Chuo-ku, Tokyo 104-0045, Japan; ^2^Department of Urology, Graduate School of Medical Sciences, Kyushu University, 3-1-1 Maidashi, Higashi-ku, Fukuoka 812-8582, Japan; ^3^New Frontiers Research Laboratories, Toray Industries, Inc., 10-1 Tebiro, Kanagawa, Kamakura 248-8555, Japan; ^4^Bio Science Department, Research and Development Center, Mitsui Knowledge Industry Co., Ltd., 2-7-14 Higashinakano, Nakano-Ku, Tokyo 164-8555, Japan; ^5^Department of Anatomic Pathology, Graduate School of Medical Sciences, Kyushu University, 3-1-1 Maidashi, Higashi-ku, Fukuoka 812-8582, Japan

## Abstract

Serum prostate-specific antigen (PSA) levels ranging from 4 to 10 ng/mL is considered a diagnostic gray zone for detecting prostate cancer because biopsies reveal no evidence of cancer in 75% of these subjects. Our goal was to discover a new highly specific biomarker for prostate cancer by analyzing plasma proteins using a proteomic technique. Enriched plasma proteins from 25 prostate cancer patients and 15 healthy controls were analyzed using a label-free quantitative shotgun proteomics platform called 2DICAL (2-dimensional image converted analysis of liquid chromatography and mass spectrometry) and candidate biomarkers were searched. Among the 40,678 identified mass spectrum (MS) peaks, 117 peaks significantly differed between prostate cancer patients and healthy controls. Ten peaks matched carbonic anhydrase I (CAI) by tandem MS. Independent immunological assays revealed that plasma CAI levels in 54 prostate cancer patients were significantly higher than those in 60 healthy controls (*P* = 0.022, Mann-Whitney *U* test). In the PSA gray-zone group, the discrimination rate of prostate cancer patients increased by considering plasma CAI levels. CAI can potentially serve as a valuable plasma biomarker and the combination of PSA and CAI may have great advantages for diagnosing prostate cancer in patients with gray-zone PSA level.

## 1. Introduction

Prostate cancer is the most common malignancy in the United State. In 2009, 192,280 men were estimated to have been diagnosed with prostate cancer, and 27,360 of these patients died in the United States [1]. The prostate-specific antigen (PSA) is used for the detection of prostate cancer in daily practice, but its diagnostic reliability is hampered by its low specificity. Thus, serum PSA levels ranging from 4 to 10 ng/mL are called the “gray zone” in which it is very difficult to discriminate between patients with prostate cancer and those with benign prostatic hyperplasia (BPH), prostatitis, or normal prostate. Furthermore, among the patients with serum PSA levels between 4 to 10 ng/mL, only 25% will be found to have prostate cancer [2]. Serum PSA levels can also increase in prostatitis, [3, 4] and approximately 20%–30% of prostate cancers are missed when the cut-off value is set to 4 ng/mL [5–7]. The false negative rate in the first biopsy is estimated between 12% and 32% [8, 9], and a large population of men with chronically high serum PSA levels undergo repeated biopsies to eliminate the possibility of prostate cancer [3, 4].

Our quantitative label-free shotgun proteomics analysis system, called 2-dimensional image converted analysis of liquid chromatography and mass spectrometry (2DICAL), can accurately align different liquid chromatography-mass spectrometry (LC-MS) data sets, enabling rapid comparison of a statistically sufficient number of clinical samples [10–16]. 2DICAL has a characteristic of top-down proteomics in shotgun proteomics. It converts the LC-MS spectrum data into peaks on a 2-dimensional plane with axes of mass-to-charge ratio (*m*/*z*) and retention time (RT). The peaks with the same *m*/*z* and RT are compared across the samples, and statistically significant peaks are selected. Targeted tandem mass spectrometry (MS) is conducted on the selected peaks, and the peaks are annotated by sequence search programs (see Supplemental Materials available online at doi:10.5402/2012/768190). 

Here we describe the discovery of a new candidate biomarker for prostate cancer diagnosis that we uncovered using 2DICAL to compare the plasma proteomes of prostate cancer patients with those of healthy controls.

## 2. Materials and Methods

### 2.1. Clinical Samples

Plasma samples were prospectively collected at the Department of Urology and Ophthalmology, Graduate School of Medical Sciences, Kyushu University (Fukuoka, Japan), between October 2000 and January 2008 from 162 individuals, including those suffering from prostate cancer (*n* = 54), renal cell cancer (RCC; *n* = 20), prostatitis (*n* = 6), and BPH (*n* = 22) and 60 healthy individuals who had no symptom and PSA below 10 ng/mL, and those with PSA over 4 ng/mL were periodically followed in outpatient clinic with no evidence of prostate cancer. Prostate cancer patients were definitively diagnosed by prostate biopsy. Patient characteristics including age, PSA levels, Gleason score, and TNM classification are shown in Table 1. For 2DICAL analysis, we selected prostate cancer patients and age-matched healthy controls. All patients provided written informed consent authorizing the collection and use of their samples for research. The institutional ethics committee boards of the National Cancer Center Research Institute (Tokyo, Japan) and the Kyushu University reviewed and approved our protocol.

### 2.2. Sample Preparation

To exclude sampling bias, 7 mL of each patient's whole blood was collected in a tube containing ethylenediaminetetraacetic acid-2Na (Venoject II, Terumo, Japan) before surgery or first treatment. Plasma was prepared by centrifuging samples at 1,050 ×g for 10 min at 4°C. Aliquots of 1 mL were added to 1.5-mL Eppendorf tubes and frozen at −80°C until analysis. Control samples were collected and stored identically. All samples were subjected to only 1 freeze-thaw cycle. To enrich low molecular weight plasma proteins, 500 *μ*L of plasma was diluted to 4 mL by adding 25 mM ammonium bicarbonate buffer (pH 8.0), and the diluted plasma samples were processed using a hollow fiber membrane-based low molecular weight (LMW) protein enrichment device as described previously [14, 17]. The device employs multistage filtration and cascaded cross-flow processes, and the proteins smaller than a predetermined molecular weight can be separated in a fully automated operation. The solution enriched for LMW proteins was recovered for 1 h operation and the LMW proteins were digested at 37°C for 18 h with sequencing grade modified trypsin (Promega, Madison, WI).

### 2.3. LC-MS

Trypsin-digested samples were analyzed in duplicate by nanoflow high-performance liquid chromatography (NanoFrontier nLC, Hitachi High-technologies, Tokyo, Japan) connected to an electrospray ionization quadrupole time-of-flight mass spectrometer (Q-Tof Ultima, Waters, Milford, MA). MS peaks were detected, normalized, and quantified using our 2DICAL software package [10, 12]. A serial identification (ID) number was applied to each of the MS peaks detected. The reproduction of LC-MS was monitored by calculating the correlation coefficient (CC) and coefficient of variance (CV) of every measurement.

### 2.4. Protein Identification by Tandem Mass Spectrometry (MS/MS)

Peak lists were generated using the Mass Navigator software package (Mitsui Knowledge Industry, Tokyo, Japan) and searched against the SwissProt database using the Mascot software package (Matrix Science, London, UK). Search parameters used were as follows: human protein sequences were selected, trypsin was designated as the enzyme, and up to 1 missed cleavage was allowed. Mass tolerances for precursor and fragment ions were ±0.6 Da and ±0.2 Da, respectively. The score threshold was set to a Mascot score >30. If a peptide matched to multiple proteins, the protein name with the highest Mascot score was selected.

### 2.5. Western Blot Analysis

Plasma samples were analyzed by sodium dodecyl sulfate-polyacrylamide gel electrophoresis using 10%–20% (w/v) ready-made gels (Pagel; ATTO, Tokyo, Japan) with the Laemmli buffer and electroblotted to a polyvinylidene difluoride membrane (Millipore, Billerica, MA). Primary antibodies were goat polyclonal carbonic anhydrase I (CAI) antibody (Millipore, Billerica, MA) and mouse monoclonal antibody against human complement C3b-*α* (PROGEN, Heidelberg, Germany). The membrane was incubated with the primary antibodies and then with the relevant horseradish peroxidase (HRP)-conjugated anti-goat or anti-mouse IgG as described previously [18]. Blots were developed using an enhanced chemiluminescence plus detection system (GE Healthcare, Buckinghamshire, UK).

### 2.6. Enzyme-Linked Immunosorbent Assay (ELISA)

To develop sandwich ELISA, the capture antibody (rabbit polyclonal CAI antibody, Abnova, Taipei, Taiwan) was immobilized on a 96-well plate (Thermo Fisher Scientific, MA) at a final concentration of 2.5 *μ*g/mL and incubated at 4°C overnight. A mouse monoclonal CAI antibody (0.5 *μ*g/mL; Abnova, Taipei, Taiwan) was used as the detection antibody. After incubation with HRP-conjugated goat anti-mouse IgG (Vector Laboratories, Burlingame, CA) for 1 h and then with OPD solution for 10 min, absorbance was measured at 490 nm using EnSpire AlphaPLUS (PerkinElmer, Waltham, MA).

### 2.7. Cell Lines

The human prostate cancer cell line 22Rv1 was purchased from Riken BRC Cell Bank (Tsukuba, Japan) and cultured in Roswell Park Memorial Institute Medium 1640 supplemented with 10% fetal bovine serum. Normal human prostate epithelial cells (CC-3165 PrEBM) were purchased from Lonza (Basel, Switzerland). The cells were cultured at 37°C under 5% CO_2_. The culture medium was changed every 3 days. After incubation of cell lines for 48 h, the cultured conditioned media were collected, filtered through a sterile 0.22-mm filter unit, and concentrated 100-fold by freeze drying. A 10 *μ*L sample of concentrated medium was used for Western blot analysis.

### 2.8. Immunohistochemistry

Cells were seeded on BioCoat collagen I-coated 8-well culture slides (Becton Dickinson Labware, Bedford, MA) and cultured overnight. The cells were washed twice with PBS (pH 7.2) and fixed with 4% paraformaldehyde in PBS for 20 min at room temperature. Fixed cells were permeabilized with 0.1% Triton in PBS for 5 min, washed 3 times with PBS, blocked for 30 min with 5% normal donkey serum (Chemicon International, Inc., Temecula, CA) in PBS, and stained with the indicated primary antibodies diluted in 1% normal donkey serum overnight at room temperature. After washing 3 times with PBS, fluorophore-conjugated secondary antibodies were applied for 1 h at room temperature and washed with PBS. Alexa Fluor 568-phalloidin was used to visualize the actin cytoskeleton. The slides were mounted with Vectashield antifade reagent (Vector Laboratories), covered with glass coverslips, and observed under Zeiss LSM510 fluorescence microscope equipped with 488/514-nm argon and 543-nm helium-neon lasers.

To stain human tissues, 10 radical prostatectomy specimens were selected with five high and five low plasma CAI concentrations. Sections (4 *μ*m) from 10% formalin-fixed paraffin-embedded material were deparaffinized in xylene and rehydrated in ethanol. Endogenous peroxidase activity was blocked by methanol containing 0.3% hydrogen peroxidase for 30 min. Microwave heating was used for antigen retrieval. The sections were incubated at 4°C overnight with a primary antibody and then incubated with a second antibody for 40 min at room temperature. The reaction products were visualized using diaminobenzidine tetrahydrochloride and counterstained with hematoxylin.

### 2.9. Statistical Analysis

Statistical significance of intergroup differences was assessed by the Welch's *t*-test and Mann-Whitney *U* test. Area under the receiver operating characteristic (ROC) curve (AUC) was calculated for each marker to evaluate its diagnostic significance. A composite index of 2 markers was generated using the results of multivariate logistic regression analysis, which also enabled the calculation of sensitivity, specificity, and ROC curve. Statistical analyses were performed using an open-source statistical language R with the optional module design package.

## 3. Results

### 3.1. Plasma Biomarker Discovery by Quantitative MS

**Figure 1:**
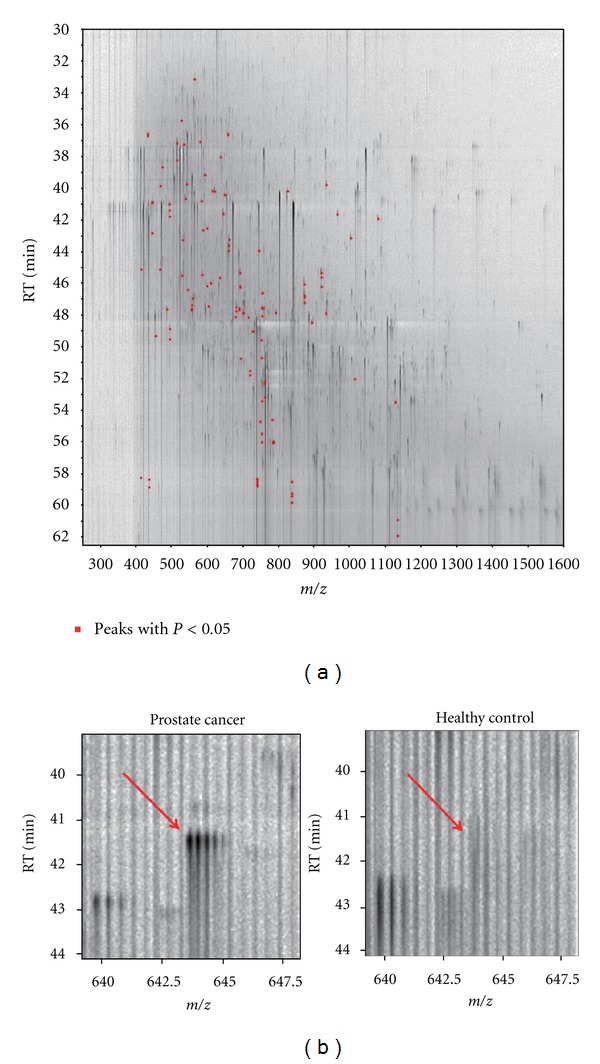
(a) Two-dimensional display of all MS peaks. The 117 MS peaks whose mean intensities significantly differed between prostate cancer patients and healthy controls (*P* < 0.05, Welch's *t*-test) are highlighted in red. RT: retention time. (b) 2DICAL images of peak ID 396 in a representative prostate cancer patient (left) and a healthy control (right).

**Figure 2:**
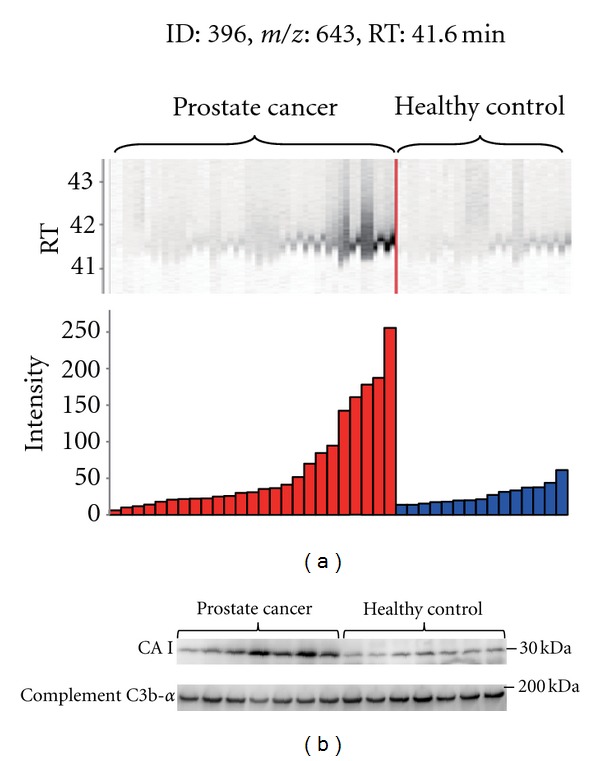
(a) MS peaks of peak ID 396 in triplicate LC-MS runs (25 with prostate cancer (left) and 15 with healthy controls (right)) aligned along LC RT. Columns represent the mean intensities of triplicate runs. (b) Immunoblotting of CAI and complement C3b-*α* (loading control) for 7 prostate cancer patients selected from the most intense MS peaks and 7 healthy controls from the least intense peaks.

To identify a diagnostic biomarker for prostate cancer patients we compared the plasma proteins of 25 prostate cancer patients with those of 15 healthy controls using 2DICAL (Table 1). Among 40,678 independent MS peaks detected within 250–1, 600 *m*/*z*, and 30–62.5 min, we found 117 peaks showing significant differences between prostate cancer patients and healthy controls (*P* < 0.05, Welch's *t*-test).  Figure 1(a) shows a representative 2-dimensional view of all MS peaks displayed with *m*/*z* along the *x*-axis and LC retention time along the *y*-axis. The 117 MS peaks whose expression levels differed significantly between cancer patients and healthy controls are highlighted in red. MS/MS spectra acquired from these 117 MS peaks matched 4 proteins in the database with Mascot scores of >30. Ten peaks matched amino acid sequences of CAI (Table 2). The CAI-derived peak (ID 396) that clearly differed between cancer patients and healthy controls is shown as a representative peak (Figure 1(b)). The intensity distribution of peak ID 396 was different between prostate cancer patients (left) and healthy controls (right) (Figure 2(a)). Immunoblotting with a CAI probe confirmed the results of the 2DICAL findings (Figure 2(b)).

### 3.2. Large-Scale Validation of Plasma CAI by ELISA

**Figure 3:**
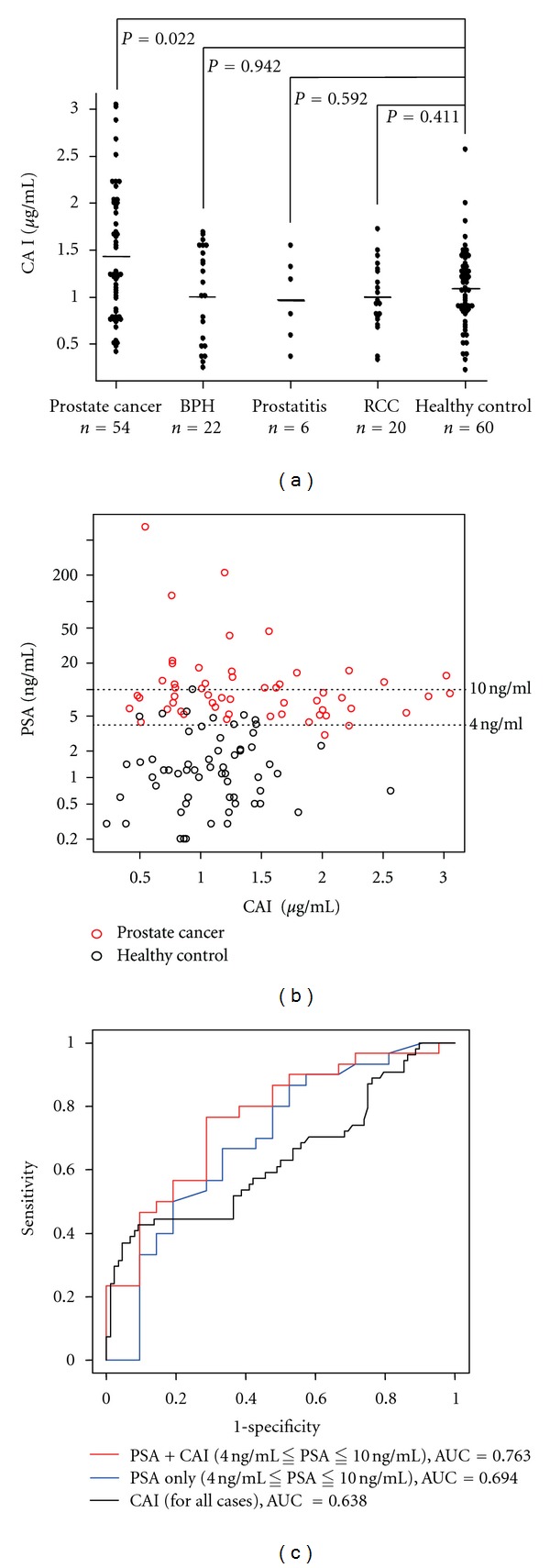
(a) Plasma CAI levels in patients with prostate cancer (*n* = 54), BPH (*n* = 22), prostatitis (*n* = 6), and RCC (*n* = 20) and healthy controls (*n* = 60) were 1.43 ± 0.69, 1.03 ± 0.51, 0.98 ± 0.46, 0.99 ± 0.36, and 1.09 ± 1.82 *μ*g/mL (mean ± SD), respectively. There was a significant difference between prostate cancer patients and healthy controls (*P* = 0.020, Mann-Whitney *U* test). Horizontal lines represented the average levels. (b) Scatter plot correlating PSA and CAI levels in prostate cancer patients (red) and healthy controls (black). The dotted lines were added to indicate the PSA gray zone. Serum PSA levels were measured using the Tandem-R kit before the first treatment in each patient. (c) ROC curve of PSA plus CAI (red line) and PSA alone (blue line) confined to the cases with PSA levels in the gray zones. ROC curves were created using a composite index of the 2 markers generated from the results of multivariate logistic regression analysis. As a reference, ROC curves of CAI (black line) for all cases were included.

To further validate plasma CAI levels in prostate cancer patients determined using 2DICAL, we performed ELISA to quantify plasma CAI levels in numerous plasma samples. The plasma samples were derived from patients suffering from prostate cancer (*n* = 54), prostatitis (*n* = 6), BPH (*n* = 22), and RCC (*n* = 20) as well as from healthy controls (*n* = 60). Plasma CAI levels were significantly different between prostate cancer patients and healthy controls (*P* = 0.022, Mann-Whitney *U* test). Plasma CAI levels of patients with BPH or RCC did not show significant differences from healthy controls. Plasma CAI levels of prostate cancer patients were clearly higher than those of any other patients (Figure 3(a)).

### 3.3. Combination of Plasma CAI Levels and PSA Assays

To determine whether plasma CAI levels and PSA assays together would be useful for diagnosing prostate cancer, CAI and PSA levels from the same cases were compared (Figure 3(b)). Pearson's CC between them was −0.106, which meant that these 2 proteins had different vectors in blood concentration. From the distribution data in the plot, CAI levels in prostate cancer patients with PSA levels of >20 ng/mL were low and CAI levels were higher in prostate cancer patients with PSA levels in the gray zone, compared to healthy controls.

To understand the diagnostic significance of CAI levels that coincided with PSA levels in the gray zone, subjects whose PSA levels were within this gray zone were investigated further. Selected cases were as follows: prostate cancer (*n* = 30), BPH (*n* = 10), prostatitis (*n* = 2), and healthy controls (*n* = 9). ROC curves for PSA alone and PSA plus CAI were generated for prostate cancer patients relative to the other cases as described in Materials and Methods (Figure 3(c)). AUCs were 0.763 and 0.694, respectively, for PSA plus CAI and PSA alone in the gray zone. PSA levels had a great discriminatory result with an AUC of 0.939 for all cases. However, PSA levels in the gray zone did not provide sufficient discriminatory power when considered alone. This indicates that CAI levels could improve the PSA assay.

### 3.4. Subcellular Localization and Secretion of CAI

**Figure 4:**
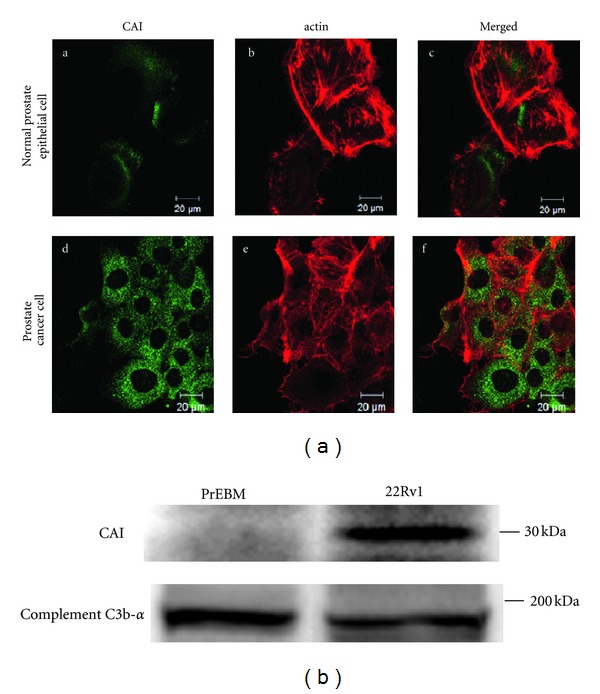
CAI expression in cell lines. (a) Immunofluorescence detection of CAI in normal prostate epithelial cells and prostate cancer cells. The cells were fixed for 1 h, permeabilized, and stained with antibodies to detect CAI. Alexa Fluor 488- or Alexa Fluor 568-phalloidin was used to visualize normal prostate epithelial cells (upper panels) and prostate cancer cells of 22Rv1 (lower panels). (b) Western blot analysis of media conditioned by the normal prostate epithelial cells and prostate cancer cells probed using a CAI antibody. C3b-*α* was used as a loading control.

The subcellular localization of CAI was investigated by immunofluorescence microscopy. CAI was hardly detected in the normal prostate epithelial cells. In comparison to actin as a cytoskeletal marker (red, Figure 4(a), b and e), immunofluorescence staining with anti-CAI (green, Figure 4(a), a and d) was observed in the cytoplasm of prostate cancer cells (merged, Figure 4(a), f). CAI was clearly detected in media harvested from 22Rv1 cells (Figure 4(b)). 

### 3.5. The Staining of Human Prostate Cancer Cells

**Figure 5:**
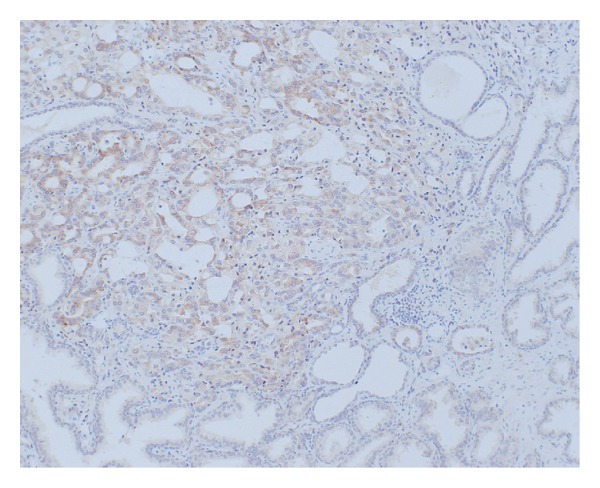
Immunohistochemical staining of CAI. CAI was strongly stained at prostate cancer (magnification ×100).

CAI was expressed in every case of prostate cancer. Cancer cells from patients with high plasma CAI concentrations tended to have stronger CAI staining than normal prostatic glands (Figure 5). 

## 4. Discussion

We took advantage of our originally developed label-free proteomic technique (2DICAL) [16] to discover a better biomarkers for prostate cancer diagnosis. We identified that CAI peptide fragments were detected at higher levels in plasma samples from prostate cancer patients than in plasma samples from healthy controls. The 2DICAL results were confirmed by Western blot analysis and ELISA using numerous plasma samples including those from patients with urological diseases other than prostate cancer. We found that the combination of CAI and PSA assays has a potential for improving the specificity of PSA assay especially for PSA levels in the gray zone. These initial results suggest that it is reasonable to vigorously pursue CAI as a potentially valuable new biomarker for prostate cancer.

The PSA assay has largely improved the detection of prostate cancer, but only approximately 25% of patients with PSA levels in the gray zone indeed have prostate cancer [19]. Despite the development of several variations of the PSA assay such as free PSA, PSA velocity, or PSA density, these methods do not substantially outperform analysis of total PSA and are not more specific [19, 20]. The reason is because healthy males are known to have PSA levels in the gray zone. Therefore, improved prostate cancer markers such as CAI are eagerly awaited to overcome these problems and enhance diagnostic specificity and sensitivity.

CAI is a zinc metalloenzyme that catalyzes the reversible hydration of carbon dioxide to bicarbonate. Sixteen CA isoforms exist in mammals [21]. Some of them are cytosolic (CAI, CAII, CAIII, CAVII, and CAXIII) catalyzing the hydration of CO_2_ to H^+^ and HCO_3_^−^, and subsequently, exporting them from the cell in exchange for Na^+^ and Cl^−^ ions [22]. CAI is a specific marker for the cytoplasm or apical cell membranes of colonic epithelial cells [22, 23] and is related to enterocyte proliferation [24]. CAI is also involved in electroneutral NaCl reabsorption and short chain fatty acid uptake [22]. The biological functions of CAs are of great interest, but CA family members are also being studied as drug targets for treating several diseases such as glaucoma, cancer, obesity, and infections [25]. 

To the best of our knowledge this is the first report to consider a correlation between CAI blood levels and prostate cancer. To verify the upregulation of plasma CAI in prostate cancer, we investigated the cell biology of prostate cancer using immunofluorescent staining to elucidate its subcellular localization, and Western blotting of culture media to determine if CAI was secreted. We demonstrated increased CAI production and secretion in prostate cancer cell lines. Stronger staining of CAI was observed in prostate cancer cells from patients with high plasma CAI concentrations compared to that in normal prostate glands. However, to clarify the mechanism of the plasma concentration change of CAI, further investigations will be needed considering the secretion mechanism of CAI from prostate cancer cells. 

Our present proteome research is new in that we found increased plasma CAI levels in prostate cancer patients. Furthermore, it indicates the possibility that CAI was produced and secreted by prostate cancer cell lines. This study may lead to clinically using CAI as a new prostate cancer marker and the combination of PSA and CAI may have great advantages for diagnosing prostate cancer in patients with gray-zone PSA levels.

## Supplementary Material

Flow chart of 2DICAL analysis: In the first step, 2DICAL discovers candidate peaks via the differential analysis of LC-MS peaks. The candidate peaks are identified by targeted tandem MS in the second step.

## Figures and Tables

**Table 1:** Clinicopathological characteristics of individuals examined in this study.

		All cases (*n* = 162)						Cases analyzed by 2DICAL (*n* = 40)		
		PCa*(*n* = 54)	BPH (*n* = 22)	Prostatitis (*n* = 6)	RCC (*n* = 20)	Healthy (*n* = 60)	*P* value	PCa* (*n* = 25)	Healthy (*n* = 15)	*P* value
Age	(mean ± SD)	66. 3 ± 7.3	72.3 ± 7.5	65.8 ± 5.9	63.8 ± 8.5	67.2 ± 8.8	0.544****	67.0 ± 7.2	65.1 ± 8.0	0.440****

Stage	I	0						0		
	II	50						22		
	III	2						2		
	IV	2						1		

TNM classification**										
	T1cN0M0	25						10		
	T2aN0M0	15						7		
	T2bN0M0	2						1		
	T2cN0M0	8						4		
	T3aN0M0	2						2		
	T4N0M1b	1						1		
	T4N1M1b	1						0		

PSA***	ng/mL									
	<4	2	11	4		51		2	12	
	4≦, ≦10	30	10	2		9	<0.001*****	14	3	<0.001*****
	10<	22	1	0		0		9	0	

Gleason score										
	5	2						1		
	6	15						7		
	7	25						12		
	8	8						4		
	9	2						1		
	10	2						0		

*Abbreviation: PCa: prostate cancer; **according to TNM Classification of Malignant Tumors (International Union Against Cancer), 6th Edition; ***measured by Tandem R kit before the first treatment; ****Welch's *t*-test; *****Fisher's exact test.

**Table 2:** Summary of protein identification by tandem mass spectrometry.

ID	*m*/*z*	RT* (min)	Charge	Normal (mean ± SD)	PCa*	*P* Values**	Mascot score	Peptide sequence	Protein description	Uniprot ID
					(mean ± SD)					
396	643.7	41.6	3	27.35 ± 13.42	63.53 ± 67.39	0.015	95.02	HDTSLKPISVSYNPATAK	Carbonic anhydrase 1	CAH1_HUMAN
310	790.9	47.8	2	24.96 ± 9.73	62.84 ± 72.61	0.016	72.71	ESISVSSEQLAQFR	Carbonic anhydrase 1	CAH1_HUMAN
983	753.0	47.5	3	16.02 ± 4.60	30.40 ± 27.50	0.017	71.62	EIINVGHSFHVNFEDNDNR	Carbonic anhydrase 1	CAH1_HUMAN
1463	719.4	51.8	2	25.20 ± 22.26	11.26 ± 2.32	0.030	66.87	GLEEELQFSLGSK	Complement C4-A	CO4A_HUMAN
1656	872.4	46.0	2	16.99 ± 5.22	25.86 ± 19.63	0.041	66.24	LYPIANGNNQSPVDIK	Carbonic anhydrase 1	CAH1_HUMAN
311	513.8	37.2	2	37.71 ± 16.18	86.33 ± 85.17	0.010	59.09	YSSLAEAASK	Carbonic anhydrase 1	CAH1_HUMAN
798	585.3	45.4	2	21.08 ± 6.68	43.99 ± 34.82	0.004	56.32	SADFTNFDPR	Carbonic anhydrase 2	CAH2_HUMAN
278	485.8	47.6	2	40.70 ± 20.28	91.91 ± 92.78	0.013	50.23	VLDALQAIK	Carbonic anhydrase 1	CAH1_HUMAN
538	493.2	41.0	2	20.19 ± 5.79	47.30 ± 52.18	0.016	44.71	GGPFSDSYR	Carbonic anhydrase 1	CAH1_HUMAN
2429	680.4	47.5	2	27.43 ± 10.68	20.70 ± 3.90	0.032	43.87	LNDLEDALQQAK	Keratin, type II cytoskeletal 1	K2C1_HUMAN
1369	714.4	48.1	3	21.16 ± 5.02	31.96 ± 19.53	0.014	43.57	YDPSLKPLSVSYDQATSLR	Carbonic anhydrase 2	CAH2_HUMAN
916	494.3	48.9	2	12.19 ± 4.64	31.68 ± 32.02	0.006	39.1	VVDVLDSIK	Carbonic anhydrase 2	CAH2_HUMAN
747	920.8	45.3	3	14.00 ± 4.82	35.85 ± 40.39	0.013	36.65	SLLSNVEGDNAVPMQHNNRPTQPLK	Carbonic anhydrase 1	CAH1_HUMAN
1596	656.3	36.6	2	9.93 ± 2.84	19.94 ± 16.30	0.006	33.83	QSPVDIDTHTAK	Carbonic anhydrase 2	CAH2_HUMAN
3781	826.1	40.2	3	4.72 ± 0.94	9.10 ± 9.25	0.027	32.29	TSETKHDTSLKPISVSYNPATAK	Carbonic anhydrase 1	CAH1_HUMAN
1199	538.3	40.7	3	19.72 ± 3.65	30.15 ± 23.16	0.036	30.33	YSAELHVAHWNSAK	Carbonic anhydrase 1	CAH1_HUMAN

*Abbreviation: RT: retention time; PCa: prostate cancer

**Welch's *t*-test.
