# Supplementary material for: Carbonic Anhydrase I as a New Plasma Biomarker for Prostate Cancer
Source: ISRN Oncol. 2012 Nov 19;2012:768190. doi: 10.5402/2012/768190 (PMC3506895; doi:10.5402/2012/768190)
Supplement: Supplementary file 1 — Flow chart of 2DICAL analysis: In the first step, 2DICAL discovers candidate peaks via the differential analysis of LC-MS peaks. The candidate peaks are identified by targeted tandem MS in the second step. [file 768190.f1.pdf]

Flow chart of 2DICAL analysis

Fig. S1

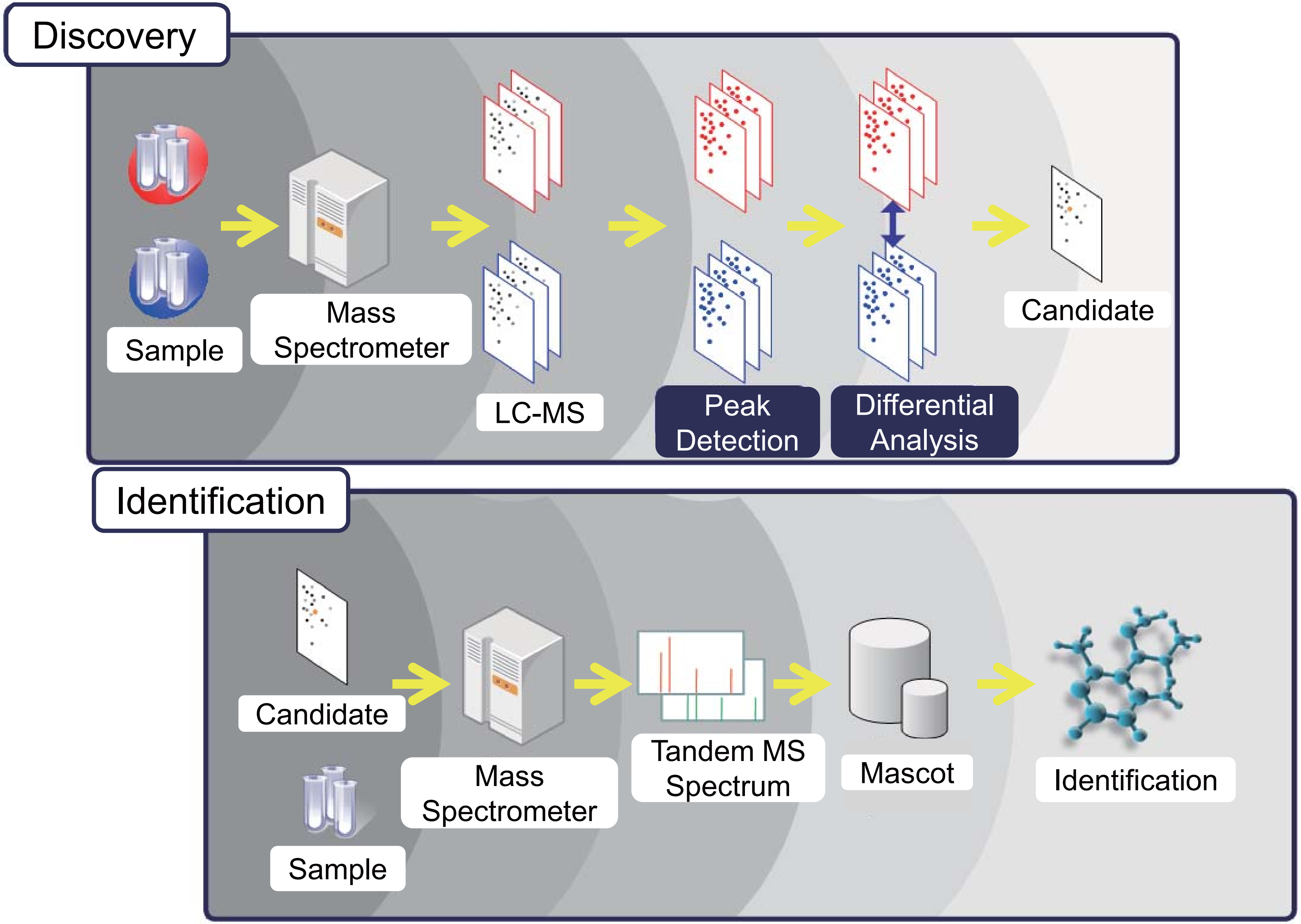

In the first step, 2DICAL discovers candidate peaks via the differential analysis of LC-MS peaks. The candidate peaks are identified by targeted tandem MS in the second step.
